# Somatoform disorders. Models of personification oriented therapy

**DOI:** 10.1192/j.eurpsy.2022.990

**Published:** 2022-09-01

**Authors:** O. Kudinova, B. Mykhaylov, T. Chorna

**Affiliations:** 1 Kharkiv Medical Academy Postgraduate Education, Psychotherapy, Kharkiv, Ukraine; 2 National University of Health Care named Shupik, Psychiatry, Kyiv, Ukraine; 3 National Pirogov Memorial Medical University, Psychiatry, Vinnytsya, Ukraine

**Keywords:** Somatoform disorders, Personal perception, Personification psychotherapy

## Abstract

**Introduction:**

Contemporary in Ukraine the special priority has been the somatoform disorders increase. The most significant complications belong to the patient’s self-evaluation of the influence of the disease on their social functioning, influence essential part of the self-evaluation of the disease and the important point of therapeutic personality accomodate intervention.

**Objectives:**

Develop the stages of personalized models of psychotherapy

**Methods:**

On the basis of the examined 270 somatoform disorders patients and 190 ischemic cardial disease patients we have elaborated a formal test that allows to evaluate quantitatively the influents of the disease on various spheres of patients´ social status.

**Results:**

It was absolutely unrespectable the common for ischemic cardial and somatoform disorders patients rise of significance of personal individual, common life factors in cases of aggravation of the main disease course. We created the personification accomodate psychotherapy system consist with CBT, suggestive and autosuggestive implementations. Elucidation of peculiarity of personal perception of the disease served as basis of elaboration of psychotherapy system, consulting, psychological support for patients with high-effectiveness 1,5 - 3 years catamnes in 85% patients.

**Conclusions:**

The retrospective results showed the necessity the personification oriented models of psychotherapy, parted on stages. On the first stage - sedative-adapting the receptions of CBT and suggestive psychotherapy are used. There is group therapy on second stage. On the third stage-supportive elements of the autogenic training mastered.

**Disclosure:**

No significant relationships.

